# Helping oxytocin deliver: considerations in the development of oxytocin-based therapeutics for brain disorders

**DOI:** 10.3389/fnins.2013.00035

**Published:** 2013-03-15

**Authors:** K. MacDonald, D. Feifel

**Affiliations:** Department of Psychiatry, University of California, San DiegoSan Diego, CA, USA

**Keywords:** oxytocin, pharmacology, humans, intranasal administration, psychiatry, drug development

## Abstract

Concerns regarding a drought in psychopharmacology have risen from many quarters. From one perspective, the wellspring of bedrock medications for anxiety disorders, depression, and schizophrenia was serendipitously discovered over 30 year ago, the swell of pharmaceutical investment in drug discovery has receded, and the pipeline's flow of medications with unique mechanisms of action (i.e., glutamatergic agents, CRF antagonists) has slowed to a trickle. Might oxytocin (OT)-based therapeutics be an oasis? Though a large basic science literature and a slowly increasing number of studies in human diseases support this hope, the bulk of extant OT studies in humans are single-dose studies on normals, and do not directly relate to improvements in human brain-based diseases. Instead, these studies have left us with a field pregnant with therapeutic possibilities, but barren of definitive treatments. In this clinically oriented review, we discuss the extant OT literature with an eye toward helping OT deliver on its promise as a therapeutic agent. To this end, we identify 10 key questions that we believe future OT research should address. From this overview, several conclusions are clear: (1) the OT system represents an extremely promising target for novel CNS drug development; (2) there is a pressing need for rigorous, randomized controlled clinical trials targeting actual patients; and (3) in order to inform the design and execution of these vital trials, we need further translational studies addressing the questions posed in this review. Looking forward, we extend a cautious hope that the next decade of OT research will birth OT-targeted treatments that can truly deliver on this system's therapeutic potential.

## Oxytocin: tool or treatment?

Over the last several decades, the nonapeptide oxytocin (OT) has been cast in two roles on the stage of human neuroscience. First and most dramatic has been its remarkable and ever-expanding role as a powerful mediator of myriad aspects of our uniquely social brains (MacDonald and MacDonald, 2010). Beginning with its evolutionary origin 10,000 years ago—when the progenitor nonapeptide vasotocin was orchestrating decision-making in marine animals (Grimmelikhuijzen and Hauser, 2012)—a series of vital advances have ratcheted forward our understanding of this vital central system. These include its initial discovery as a uterotonic component of pituitary extract over a century ago (Dale, 1906); the concept (novel at the time) of neurosecretion, the “glandular activity” of hormone-secreting neurons (Scharrer and Scharrer, 1945); the vital technique of immunoflourescent visualization of OT-producing neurons (Swaab et al., 1975) which allowed the subsequent histological characterization of the human central OT system (Loup et al., 1991); the more recent sequencing and synthesis of the peptide (Du Vigneaud, 1956), and the gene for the receptor (Kimura et al., 1992); and finally, increasingly sophisticated translational research using techniques like gene knockout and optogenetic manipulation of specific central circuits (Stoop, 2012). Each of these progressive steps has allowed us to ask and answer increasingly specific questions about the nature of the central OT system and its contribution to the bonded, social nature we share with fellow mammals. The culmination of several decades of sophisticated translational neuroscience research has been a decade-long groundswell of human studies which have ensconced OT and its sister nanopeptide vasopressin with testosterone, estrogen, and cortisol in the pantheon of centrally active hormones critical to understanding human behavior.

OT has also been cast for a second, as-yet unfulfilled role as a therapeutic tool to ameliorate suffering from brain-based disease. Promise notwithstanding, its performance here has been a bit more pedestrian. Although it has been safely used for decades in obstetrics to induce and augment labor, the suggestion that OT may have therapeutic value in the treatment of a host of brain-based conditions (addiction, anxiety, autism, mood disorders, and schizophrenia) has not for the most part been bolstered by clinical investigations with meaningful therapeutic endpoints. More precisely, despite an enormous amount of anticipation that OT's effects in preclinical studies can be translated into OT-based treatments for psychiatric disorders, few studies have actually delivered OT as a bona-fide therapeutic agent, using chronic daily dosing, targeting core symptoms of specific disease states, and assessing safety, tolerability, and clinical outcomes. From a clinical perspective, the current portfolio of OT research is flush with more therapeutic hints than help, and exogenous OT has—thus far—acted more decisively in its role as a pharmacological probe than a therapeutic palliative.

These contrasting roles come into apposition at a time in the history of psychiatric therapeutics which some have called a “crisis” (Fibiger, 2012). To whit, despite significant advances in our understanding of the brain bases of human psychiatric disease, our abilities to reduce suffering and restore function in psychiatric disease remains woefully inadequate (Holma et al., 2008; Lieberman and Stroup, 2011; Volkow and Skolnick, 2012). Many or most of the foundational therapeutic medications in our psychiatric armamentarium (i.e., antidepressants, antipsychotics) were discovered serendipitously decades ago. Several promising new therapeutic classes of drugs for CNS conditions have failed to pass late-stage drug development. Stymied by these repeated, costly product failures, many pharmaceutical companies have abjured investing in this therapeutic arena. These pharmaceutical realities stand in stark contrast to the significant prevalence and toll of brain-based diseases. Though these setbacks pose a challenge to drug development, we maintain optimism that OT-based therapeutics may provide relief, though as discussed below, development of OT-targeted therapeutics has its own challenges.

In this review, we approach OT from the perspective of researcher-clinicians interested in the development of OT-targeted pharmaceuticals for the abridgement of human psychiatric disease. Given intense interest in this molecule and the ever-mushrooming literature, this review has a necessarily limited scope. Herein, we constrain our discussion to arenas of significant, direct relevance to the development of OT-based therapeutics, and direct interested readers to several recent, well-referenced reviews on other vital aspects of OT including details of its neurophysiology and interaction with arginine vasopressin (AVP) (Stoop, 2012), implications of genetic variations in the OT receptor (OTR; Ebstein et al., 2012; Kumsta and Heinrichs, 2013), and OT's role in human development (Gordon et al., 2011; Feldman, 2012).

## Brain disorders for which oxytocin may have therapeutic efficacy

As mentioned above, as the result of its revealed effects on behavior and brain processes observed in both animal studies and translational studies in humans, OT has been proposed as a potential treatment for a wide range of brain disorders: addiction, anxiety disorders, autism-spectrum and other developmental disorders, borderline personality disorder, mood disorders, and schizophrenia. For a more complete background on OT's preclinical profile and the justification for these therapeutic speculations see the following recent, extensive reviews (Slattery and Neumann, 2010; MacDonald and Feifel, 2012a; McGregor and Bowen, 2012; Modi and Young, 2012; Neumann and Landgraf, 2012). As of November 2012, we note ongoing treatment trials of intranasal (IN) OT in autism, schizophrenia and schizoaffective disorder, frontotemporal dementia, major depressive disorder and treatment resistant depression, post-traumatic stress disorder, borderline personality disorder, and drug dependence (i.e., alcohol, marijuana) (www.clinicaltrials.gov). Therewith, we anticipate that the next several years will show a corresponding increase in actual clinical data. To date, however, there have been a relatively limited number of patient-targeted clinical OT trials, with the majority being single-dose (Table 1). Given the limited number of studies that have been conducted evaluating OT's potential as a bona-fide treatment for clinical brain disorders, one could conclude that almost all of the much-anticipated therapeutic potential of this neuropeptide remains to be proven.

**Table 1:** **Studies using oxytocin in patients with brain-based illness**.

**References**	**Population**	***N*, sex, age (if < 18)**	**Parameter studied**	**Dosing**	**Findings**
**SINGLE-DOSE TRIALS**					
Hollander et al., 2003	Autism	14 M, 1 F	Repetitive behaviors	Up to 70 U/h IV	OT caused a significant reduction in repetitive behaviors.
Hollander et al., 2007	Autism	15 M	Affective speech comprehension	Up to 70 U/h IV	OT subjects showed improvements in affective speech comprehension and ability to accurately assign emotional significance to speech intonation.
Andari et al., 2010	Autism and Asperger's	11 M, 2 F	Social behavior in a multiplayer game and eye contact	24 IU OT	(1) OT caused stronger interactions with cooperative partner, increased trust and preference, and increased gaze to eyes. (2) IN OT elevated OT plasma levels, but less than controls.
Guastella et al., 2010	Autism and Asperger's	16 M, age 12–19	Social cognition: RMET performance	18 IU (Age 12–15); 24 IU (Age 16–19)	Improved performance on the RMET.
Bartz et al., 2011a	Borderline personality disorder	10 F, 4 M	Neuroeconomic trust game	40 IU	OT impeded trust and prosocial behavior, moderated by attachment anxiety and avoidance.
Simeon et al., 2011	Borderline personality disorder	6 F, 8 M	Post stressor subjective mood, cortisol response	40 IU	OT attenuated subjective post-stressor dysphoria, and caused a trend to decreased cortisol. Results moderated by trauma, self-esteem, and attachment style.
Hall et al., 2012	Fragile-X syndrome	8 M, age 13–28	Eye gaze frequency, heart rate, heart rate variability, cortisol	24–48 IU	Eye-gaze improved with 24 IU; cortisol decreased with 48 IU.
Pincus et al., 2010	Major depressive disorder	8 F	Reaction time and brain responses (fMRI) to RMET	40 IU	(1) Compared with controls, depressed patients doing the RMET activated higher order cognitive areas and insula with OT. (2) OT caused slower reaction time in depressed group.
MacDonald et al., 2011b	Major depressive disorder	17 M	Social cognition (RMET)	40 IU	OT improved RMET scores.
Pitman et al., 1993	Post-traumatic stress disorder	43 M	Physiologic responses (HR, GSR, facial EMG) to personal trauma prompts	20 IU	OT subjects had the lowest mean physiologic responses to personal combat imagery prompts, verses placebo and IN AVP-treated subjects.
Mah et al., 2013	Postnatal depression	25 F	Self-reported mood and ratings of mother-infant relationship	24 IU	OT-treated mothers were sadder and described babies as more difficult, but described the relationship quality as more positive.
Averbeck et al., 2011	Schizophrenia	(1) 24 M, 6F (2) 21 M	Emotion recognition (hexagon emotion discrimination test)	24 IU	Patients had deficit in emotion recognition compared to controls, and OT improved ability of patients to recognize most emotions.
Goldman et al., 2011	Schizophrenia	7 M, 6 F	Judgment of presence and intensity of facial emotions	10 IU, 20 IU	10 IU dose caused decreased emotion recognition; 20 IU dose improved emotion recognition.
Labuschagne et al., 2010	Social anxiety disorder (generalized)	18 M	Brain responses (fMRI) to emotional face matching task with fearful, angry, happy faces	24 IU	Patients exhibited bilateral amygdala hyperactivity to fearful faces; OT normalized this effect.
Labuschagne et al., 2011	Social anxiety disorder (generalized)	18 M	Brain responses (fMRI) to emotional face matching task of happy and sad (vs. neutral) faces	24 IU	Patients had heightened activity to sad faces in medial prefrontal cortex and anterior cingulate cortex; OT reduced this hyperactivity.
Guastella et al., 2009	Social anxiety disorder	25 M	Self-rated aspects of social anxiety, speech performance and appearance.	24 IU	OT-treated subjects demonstrated improved self-evaluation of appearance and speech performance; these benefits did not generalize into a sustained positive effect over exposure therapy alone.
**MULTIPLE-DOSE TRIALS/CASE REPORTS**					
Pedersen et al., 2013	Alcohol dependence	9 M, 2 F	Alcohol withdrawal scores, lorazepam use	24 IU twice-daily for 3 days	OT-treated patients required less lorazepam, had lower alcohol withdrawal scores, and lower subjective distress.
Kosaka et al., 2012^*^	Autism	1 F, age 16	Social interaction, aberrant behavior checklist, CGI	6 months of IN OT (8 IU daily)	Improvement in social interaction and communication, irritability and aggressive behavior.
Anagnostou et al., 2012	Autism-spectrum disorder	16 M, 3 F	Social function/cognition, repetitive behaviors, social responsiveness, RMET, YBOCS, WHOQOL	6 w of 24 IU BID	Though no significant changes on primary endpoints; RMET, repetitive behaviors, and QOL improved.
Feifel et al., 2011	Generalized anxiety disorder	7 M, 6 F	HAM-A	20 IU twice-daily for 1 w, then 40 IU twice-daily for 2 weeks	Males showed significant decrease in anxiety at week 2, females showed trend increase in anxiety, with trend significance drug × gender interaction.
Ohlsson et al., 2005^#^	Irritable bowel syndrome	49 F	Constipation and associated subjective parameters	40 IU twice daily for 13 weeks	OT caused slightly improved mood, abdominal pain and discomfort.
Scantamburlo et al., 2011^*^	Major depressive disorder	1 M	HAM-D, STAI, Q-LES-Q	Up to 36 IU over several weeks	Adjunctive OT improved depressive and anxiety symptoms and quality of life over the course of weeks.
den Boer and Westenberg, 1992	Obsessive compulsive disorder	3 M, 9 F	Obsessions and compulsions	(1) 18 IU IN for 6 weeks (dosed four times daily) (2) 2 M treated with 54 IU	No effect on symptoms.
Epperson et al., 1996a	Obsessive compulsive disorder	3 F, 4 M	Obsessive compulsive disorder symptoms, anxiety, mood and memory	160 IU or 320 IU IN (divided four times daily) for 1 week	No change in obsessive-compulsive disorder symptoms. OT subjects had a statistically significant improvement in BDI.
Bujanow, 1972	Schizophrenia	Not mentioned	Underspecified	10 IU-15 IU IV; 20 IU-25 IU IM daily 6–10 injections	OT induced “rapid therapeutic effects” and “hospitalizations were prevented.”
(1) Feifel et al., 2010 (2) Feifel et al., 2012a	Schizophrenia	12 M, 3 F	(1) PANSS, CGI, side effects (2) verbal memory	20 IU twice-daily for 1 week, 40 IU twice-daily for 2 weeks	(1) OT improved PANSS, CGI at 3 w time point (2) OT caused improved verbal memory.
Pedersen et al., 2011	Schizophrenia	17 M, 3 F	PANSS, social cognition	24 IU twice-daily for 2 weeks.	OT improved PANSS scores, and social cognition.
Modabbernia et al., 2013	Schizophrenia	33 M, 7 F	PANSS	20 IU twice-daily for 1 week, then 40 IU twice-daily for 8 weeks total	OT improved PANSS total, positive and negative scales by week 4. Effects on positive symptoms was more clinically robust.
Bakharev et al., 1984	Schizophrenia	27 M	Subsets of schizophrenia symptoms (not a standardized scale)	10 “active units” IV or IN twice-daily × 7 days every other week for 2 weeks	Improvements in self and clinician-rated “asthenodepressive, apathodepressive, hypochondriac symptoms” compared with conventional antipsychotic agents.
MacDonald and Feifel, 2012b^*^	Social anxiety disorder	1 M	Social anxiety symptoms, sexual function	20 IU twice daily over several weeks	Improvement in several areas of sexual function, though no benefit in social avoidance or anxiety.
Epperson et al., 1996b^*^	Trichotillomania	2 F	Trichotillomania symptoms	160 IU (divided four times daily) for 1 week	No difference in trichotillomania symptoms.

* Case reports.

# Though not in a psychiatric population per se, the length of this study and effect on mood warranted inclusion.

Abbreviations: *ASD* *autistic spectrum disorder* *AVP* *arginine vasopressin* *BDI* *Beck depression inventory* *CGI* *clinical global impression scale* *EMG* *electromyography* *F* *female* *fMRI* *functional magnetic resonance imagery* *GSR* *galvanic skin response* *HAM-A* *Hamilton rating scale for anxiety* *HAM-D* *Hamilton rating scale for depression* *HR* *heart rate* *IM* *intramuscular* *IN* *intranasal* *IU* *international units* *IV* *intravenous* *M* *male* *OT* *oxytocin* *PANSS* *positive and negative syndrome scale* *Q-LES-Q* *quality of life enjoyment and satisfaction questionnaire* *RMET* *reading the mind in the eyes test* *STAI* *state-trait anxiety Inventory* *YBOCS* *Yale Brown obsessive compulsive scale* *WHOQOL* *world health organization quality of life scale.*

## Important questions for developing oxytocin-targeted therapeutics

Most preclinical and translational studies conducted to date—as well as single-dose studies in normals—attempt to answer the question: “what does OT do?” A pragmatic, treatment-oriented clinician may wish to restate the question, asking: “what does OT do when used as drug?” More specifically, “what effects does OT have when given chronically to patients with psychiatric illness?” Sadly, in spite of a decade of high-profile studies, we would be hard-pressed to answer this question for the majority of putative indications. Delving deeper, three-linked facts make this clinically oriented query even more incisive: (1) as mentioned, the vast majority of published OT studies are single-dose studies in normals; (2) in normals, the single-dose effects of lifesaving psychiatric medications [i.e., antipsychotics, serotonin reuptake inhibitors (SSRIs)] are often either negligible (Harmer et al., 2003; Murphy et al., 2009) or aversive (Belmaker and Wald, 1977; Harmer et al., 2008); (3) in psychiatric patients, the short-term effects of some medications are the opposite of their effect when given chronically [i.e., short-term anxiogenesis with SSRIs (Kent et al., 1998)]. As such, though in some cases single-dose effects in normals can be linked to longer-term benefits in patient populations [i.e., enhancement of emotional processing as a biomarker for antidepressant activity (Harmer et al., 2009a; Tranter et al., 2009)], we should be circumspect when extrapolating too directly from these studies to the effects of chronic dosing in psychiatrically ill samples. For all these reasons, multi-week, daily dose, randomized placebo-controlled trials—the mainstay for evaluating the therapeutic efficacy and safety of investigational psychotropic drugs—are needed to advance the field from the stage of optimistic speculation into the realm where definitive verdicts can be obtained. Only then will we be able to decisively answer the vital question asked by our treatment-seeking clinician.

Beyond these sorely needed proof-of-concept clinical trials, the development of OT-targeted therapeutics for CNS disease faces a significant number of challenges. In the sections below—in the form of 10 questions—we attempt to identify and describe them (Table 2). En toto, these questions span a wide spectrum of issues that need to be addressed in order to complete the bench-to-bedside arc with OT. Of note, the question of the role of several important individual factors (variations in the OT and CD38 receptor, sex, and early experience) in clinical response to OT is covered in an accompanying mini-review (MacDonald, 2012) in this special section.

**Table 2:** **Ten questions for the development of oxytocin-targeted therapeutics for brain disorders**.

1.	How do acute and chronic oxytocin administration differ?
2.	How do oxytocin's therapeutic-like effects in healthy subjects translate to patient with brain disorders?
3.	How do oxytocin's therapeutically relevant effects differ in men and women?
4.	What is the optimal therapeutic dose range for oxytocin?
5.	What is oxytocin's optimal therapeutic dosing schedule?
6.	Can native oxytocin be improved upon?
7.	Is intranasal delivery of oxytocin the optimal route?
8.	What is the role of vasopressin receptors in oxytocin's effects?
9.	Monotherapy vs. augmentation: can oxytocin treat on its own or is it better suited to augment other established treatments?
10.	Are there identifiable biomarkers for oxytocin's therapeutic effects?

### How does acute and chronic oxytocin administration differ?

The vast majority of published studies of IN OT—even those done in patient samples (Table 1)—have used only a single-application dosing paradigm. In stark contrast, almost all treatments for the most debilitating brain disorders are delivered chronically, with most achieving their maximal clinical effects after weeks of daily administration. Furthermore, as intimated above, the acute and chronic effects of medications are often diametrically opposite, as seen in the case of SSRIs, which are a first-line chronic treatment for anxiety disorders, yet can cause anxiety after a single-dose (Spigset, 1999; Birkett et al., 2011). One process that contributes to the difference between acute and chronic drug administration is “tachyphylaxis” or “tolerance” in which the acute effects of a drug dissipate with repeated administration. At a cellular level, persistently stimulated receptors like the OTR may become desensitized or may be expressed in smaller numbers on cell surfaces, via several processes, including one called internalization. Notably, internalization has been demonstrated to occur with the OTR (Gimpl and Fahrenholz, 2001).

Specifically in the case of OT, basic science research supports the fact that that there are often significant differences between the effects of acute and chronic administration of OT (Kramer et al., 2003; Bowen et al., 2011; Keebaugh and Young, 2011; Bales and Perkeybile, 2012). Furthermore, though the neurobiological mechanism of certain of OT's effects (i.e., acute anxiolysis) have been carefully dissected in animal models (Viviani and Stoop, 2008; Yoshida et al., 2009; Viviani et al., 2011; Knobloch et al., 2012; Stoop, 2012, for review), the mechanism of action of therapeutic later-onset effects of chronic OT treatment—the mode of treatment most salient to human brain disorders—remains unknown.

On this latter point, the small body of research in which chronic IN OT has been given to psychiatrically ill patients indicates that like currently used medications from other classes, IN OTs antipsychotic (Feifel et al., 2010, 2012a; Pedersen et al., 2011; Modabbernia et al., 2013) and anxiolytic (Feifel et al., 2011) effects may take weeks to emerge to a clinically meaningful degree. Unfortunately for the development of OT-targeted therapeutics, then, the large number of extant IN OT studies may not speak directly to key issues relevant to the effects of chronic OT administration. As discussed below in section “What is the Role of Vasopressin Receptors in Oxytocin's Effects?” however, the discovery and development of biomarkers which track with clinical outcomes and syndromes would significantly improves the clinical gain from single-dose trials (de Oliveira et al., 2012a). Examples relevant to OT include the abovementioned single-dose antidepressant effects on emotional processing (Harmer, 2008) and the attenuation of the anxiogenic effects of CO_2_ inhalation (Bailey et al., 2007). We anticipate that future biomarker and pharmacokinetic studies in humans treated with acute and chronic OT will build on the few single-dose, functional-imaging trials in humans with psychiatric illness (Labuschagne et al., 2010, 2011; Pincus et al., 2010) to illuminate these important clinical questions.

### How do oxytocin's therapeutic-like effects in healthy subjects translate to patient with brain disorders?

**Figure 1:**
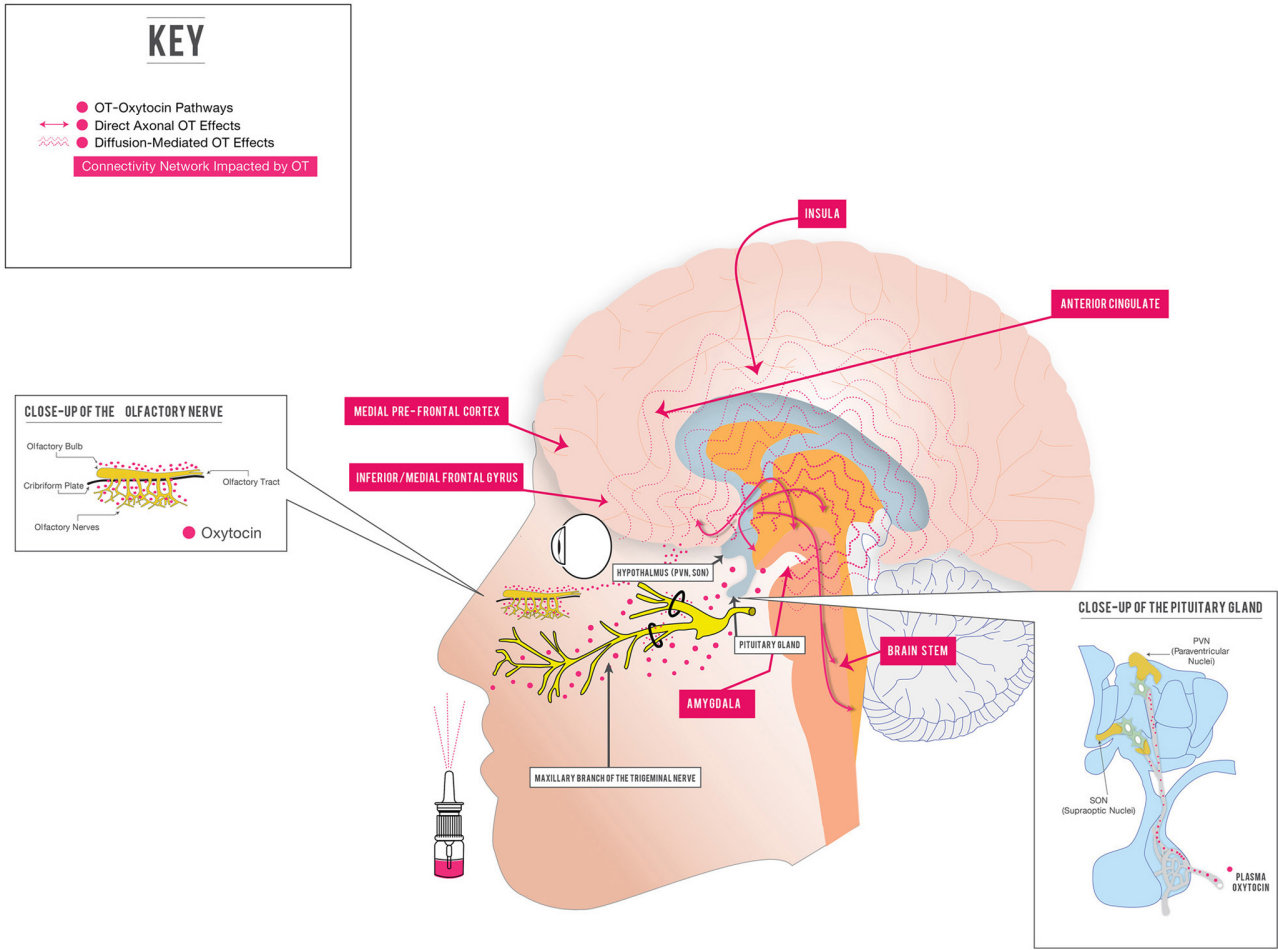
**Intranasal oxytocin: potential therapeutic regulation of brain function in psychiatric illness.** Several important aspects of intranasally delivered OT (IN OT) treatment of brain-based illness are represented. One potential way that IN OT may cross the blood-brain barrier and cause central effects is represented: directly via extraneuronal/perineuronal routes along trigeminal or olfactory nerve pathways (Thorne and Frey, 2001; Ross et al., 2004; Dhuria et al., 2010; Renner et al., 2012a,b). Other mechanisms of entry (bulk flow, lymphatic channels, intraneuronal transport, active or passive transport from vasculature) are discussed in references and the text. IN OT may cause some of its central effects by stimulating the endogenous OT system, which secretes OT into the peripheral circulation (right pullout), and has both direct, “wired” and diffusion-mediated central effects (Landgraf and Neumann, 2004; Stoop, 2012). Through these mechanisms, IN OT impacts the function of amygdala-anchored connectivity networks in normals (Kirsch et al., 2005; Sripada et al., 2013), as well as important brain regions (amygdala, insula, anterior cingulate, medial prefrontal cortex) in patients with psychiatric illness (Labuschagne et al., 2010, 2011; Pincus et al., 2010). For simplicity, not all brain areas impacted by OT are shown; see Bethlehem et al. (2012); Zink and Meyer-Lindenberg (2012) for recent, detailed reviews.

A second pharmacodynamic issue that requires more study concerns the difference between OT's effects in normal vs. psychiatrically ill samples. Just as a host of individual differences significantly influence response to OT in normal samples (MacDonald, 2012), so it is likely that OT's effects will differ between healthy and clinical populations. For example, in a functional imaging study of the effects of OT on brain activity during the reading the mind in the eyes test (RMET), OT-mediated alteration in brain activity differed significantly between untreated patients with depression and normal subjects (Pincus et al., 2010). A second, unpublished set of data from our group found that patients with depression had an anxiogenic response to single-dose IN OT given in a psychotherapy context, in contrast to acute anxiolytic effects reported in normals (Heinrichs et al., 2003; de Oliveira et al., 2012a,b). Additionally, Bartz et al. has demonstrated that many patients with borderline personality disorder have divergent responses to OT that those seen in normals, with OT-decreasing trust and cooperation (Bartz et al., 2011a). On the other hand, some acute neural responses (attenuation of amygdala activity) are seen in both patient groups (Labuschagne et al., 2010) and normals (Zink and Meyer-Lindenberg, 2012) (Figure 1). Notwithstanding these similarities, the findings from several single-dose studies indicating that the effects of OT may differ between patients with psychiatric disease and those without calls for caution when extrapolating clinical effects of OT in patients from the study of its effects in normals.

There may be, moreover, significant associations between the OT system and certain psychiatric disease states or endophenotypes. One component of this distinction is covered in an accompanying mini-review (MacDonald, 2012), which discusses the clinical import of research on variations in the OTR and the CD38 ectoenzyme. Though many of the studies in this growing literature are in normals, certain genetic variations in aspects of the OT system have been associated with disease states (Kumsta and Heinrichs, 2013). In addition to these known variations in the OT system, one could assume that certain individuals (and perhaps certain diagnostic groups) have an as-yet undocumented state of more significant functional OT deficiency akin to that found in central diabetes insipidus (DI). Specifically, in the genetic form of central DI called familial neurohypophyseal diabetes insipidus (FNDI), variations in the AVP prohormone gene (AVP-neurophysin II) on chromosome 20 result in inadequate protein folding and dimerization. These changes cause the aberrant protein to be retained in the neuron, ultimately leading to cell death of hypothalamic magnocellular neurons in the supraoptic nucleus and paraventricular nucleus (Bergeron et al., 1991; Ito and Jameson, 1997). Given endogenous AVPs role in natriuresis, these patients present clinically with symptoms of progressive functional AVP deficiency, including polyuria, and polydipsia (Robertson, 1995). Currently, more than 60 clinically relevant genetic variants of the AVP prohormone have been identified (Christensen et al., 2013). Returning to OT, then, variants of “OT deficient” animals have been created via genetic alterations of the OT gene or its receptor (Young et al., 1996; Bernatova et al., 2004; Lee et al., 2008; Nishimori et al., 2008): these animals display a range of behavioral abnormalities. As such, although no OT-related genetic syndrome akin to FNDI has yet been characterized in humans, the abovementioned findings raise the interesting question of the possibility of an “OT-deficiency syndrome” which manifests with deficits in the production and/or function of either the hormone, the receptor, or other components of the system (i.e., the ectoenzyme CD38).

### How do oxytocin's therapeutically relevant effects differ in men and women?

Given OT's intimate evolutionary involvement with reproductive function, it should not be surprising to find that is has distinct effects on the brains of males and females. Indeed, the histological structure for OT neurons is sexually dimorphic (de Vries, 2008) and sex biases in behavioral responses to OT have been frequently found in animal studies (Williams et al., 1994; Cho et al., 1999; Bales and Carter, 2003; Bales et al., 2007a). Estrogen increases OT and OTR production (Patisaul et al., 2003; Windle et al., 2006; Choleris et al., 2008), whereas testosterone promotes hypothalamic OTR-binding (Johnson et al., 1991) as well as production of AVP (Delville et al., 1996), which has many opponent actions to OT (Neumann and Landgraf, 2012).

Though the question of the meaning and measurement of OT levels is still subject to active study (see section “What is Oxytocin's Optimal Therapeutic Dosing Schedule?” below), men and women show differences in plasma OT levels (Ozsoy et al., 2009; Gordon et al., 2010; Holt-Lunstad et al., 2011; Weisman et al., 2012c), as well as sex-specific behavioral correlations with OT (Gordon et al., 2010; Zhong et al., 2012). In addition, amygdala-prefrontal cortical connectivity—which can be impacted by OT in normal subjects (Sripada et al., 2013) and anxiety patients (Labuschagne et al., 2011)—may be related in a gender-specific way to the development of anxiety and depressive disorders (Burghy et al., 2012). Furthermore, numerous studies in the growing OTR literature note sex-specific associations between genetic variants in the OTR gene and personality characteristics (Stankova et al., 2012), neural responses to emotionally salient cues (Tost et al., 2010), pair-bonding (Walum et al., 2012), hypothalamic gray matter volume (Tost et al., 2010), and empathy (Wu et al., 2012). On the other hand, several studies in this area have failed to find a sex bias (Rodrigues et al., 2009; Saphire-Bernstein et al., 2011; Feldman, 2012).

With regards to clinical studies of the effects of IN OT, a sex difference in its effects has been demonstrated in some single-dose studies (Hurlemann et al., 2010), including studies of OT's effects on the amygdala (Domes et al., 2010; Rupp et al., 2012), and interpersonal behavior (Liu et al., 2012). Again, these effects are variable: many other studies in this area have not found an effect of sex [see Bartz et al. (2011b), for review].

Focusing on the few multi-week clinical trials of OT in psychiatric populations, the three published clinical trials in schizophrenia included a disproportionate number of males (62 males treated vs. 13 females), consistent with most clinical trials of this disorder (Feifel et al., 2010, 2012a; Pedersen et al., 2011; Modabbernia et al., 2013). The number of women included in each trial was not sufficient to analyze for a sex-by-drug effect. Though schizophrenia is the clinical disorder with the largest number of separate randomized trials using IN OT, the first study to intimate a sex moderation effect of OT was a randomized, double-blind, within-subjects crossover study of OT (40 IU BID for 3 weeks) in patients with generalized anxiety disorder (GAD) (Feifel et al., 2011). This trial demonstrated a trend-level dose-by-gender effect such that males treated with OT showed a significant clinical improvement in HAM-A scores with OT, whereas females did not. En toto, the abovementioned sex differences indicate that delineation of the role of sex and sex hormones in the response to chronic OT treatment will be critical.

### What is the optimal therapeutic dose range for oxytocin?

Despite the groundswell of IN OT research, we know very little about either the optimal dose or dosing parameters of IN OT for any CNS indication, and single-dose studies, though informative, speak somewhat peripherally to this important issue. Noteworthy is that animal research indicates a discrepancy between the effects of both dose (Windle et al., 1997; Kramer et al., 2003; Bales et al., 2007b) and single vs. chronic dosing of OT (Bales and Perkeybile, 2012). Aside from optimizing therapeutic effect, dosing issues are also important in terms of side effects, given that OT has some cross-affinity with AVP receptors which mediate its potential diuretic and natriuretic effects (Gimpl and Fahrenholz, 2001), and have been noted in a single case report of high-dose IN OT (Ansseau et al., 1987). An illuminating primate study in this regard indicated that perhaps due to cross-reactivity with AVP, [which potentiates stress responses (Legros, 2001)], chronic higher-dose IN OT (200 IU) did not attenuate cortisol (ACTH) responses, whereas a lower-dose (50 IU) did (Parker et al., 2005).

Largely due to prior precedent (vs. pharmacological rationale), the majority of published human studies have tested OT in single doses in the 20–40 IU range (MacDonald et al., 2011a), though doses as low at 10 IU (Goldman et al., 2011) and as high as 160 IU daily (Epperson et al., 1996a,b) have been reported. The few chronic-dosing studies cited herein have used a dose range of 24–40 IU BID (Feifel et al., 2010, 2012a; Pedersen et al., 2011; Modabbernia et al., 2013). Importantly, very few studies have directly compared the effects of two or more doses, a standard strategy in dose-finding clinical trials.

In one the first clinical study to examine effects of multiple doses of OT in the same clinical subject, Goldman et al. demonstrated that in patients with schizophrenia, 10 IU caused a decrement in ability to identify facial emotions (due to increased false-response rate), whereas 20 IU improved emotion recognition in polydipsic relative to non-polydipsic patients (Goldman et al., 2011). This dose-related finding is interesting given that more placebo-controlled IN OT trials have been done in schizophrenia than any other indication. In a study of men with fragile X syndrome, 24 IU but not 48 IU IN OT improved eye gaze frequency, whereas 48 IU but not 24 IU decreased social-stress induced salivary cortisol levels (Hall et al., 2012). A study in normals on the effect of OT on exercise-induced increased in salivary cortisol levels demonstrated that 24 IU but not 48 IU attenuated this effect (Cardoso et al., 2012). Another related study in normal men found that IV OT (titrated to a level 10 times higher than physiologically normal baseline levels) demonstrated a linear, dose-response OT effect on ACTH and cortisol (Legros et al., 1984). Finally, a recent study documented that salivary OT levels in normal subjects remained similarly elevated for up to 7 h, regardless of which of 2 doses of IN OT patients received (16 or 24 IU) (van Ijzendoorn et al., 2012). In light of these findings suggesting that OT's effects may be dose-dependent, more studies are needed in which more than 1 dose—preferably a range of doses—are directly compared. Clearly, we need to understand whether a dose-response relationship exists regarding the effects of OT on core disease symptoms, and whether such dose-response relationships are disease specific.

### What is oxytocin's optimal therapeutic dosing schedule?

In addition to an inadequate understanding regarding the dose-response curve for most therapeutically relevant effects of OT, there is very little known regarding its optimal therapeutic-dosing frequency (e.g., once daily, twice daily, etc.). In addition to knowing the optimal dose range, dose frequency data is critical for successful design of future proof-of-concept clinical trials. Typically, dosing schedule is based upon the plasma half-life of the drug in question. However, this heuristic is likely not applicable to the CNS effects of OT, particularly OT delivered IN, given its putative direct access to the brain via this route of administration (Born et al., 2002). Specifically, whereas the plasma half-life of IV OT is less than 10 min (Mens et al., 1983), it lasts much longer in the CSF, and studies measuring plasma OT have found elevated levels of the peptide lasting more than 1 h after a single IN administration (Burri et al., 2008; Gossen et al., 2012).

Relevant here are single-dose studies measuring both salivary (Huffmeijer et al., 2012; Weisman et al., 2012a) and plasma OT levels (Burri et al., 2008; Andari et al., 2010; Domes et al., 2010; Gossen et al., 2012), which indicate that IN OT quickly elevates peripheral OT levels, and that, these levels remain above baseline for some time. In terms of mechanism, in addition to direct absorption via the nasal vasculature, it is thought that IN OT may also elevate peripheral OT levels by entering the brain and stimulating OT neurons to secrete endogenous OT from central stores into the peripheral circulation (Neumann et al., 1996) (Figure 1). This suggestion is supported by the fact that OT neurons operate in a feed-forward “bursting” mechanism, such that exogenous or endogenous OT stimulates further pulsatile OT release (Renaud et al., 1984; Rossoni et al., 2008), part of a locally regulated positive-feedback mechanism (Neumann et al., 1996). This ongoing release from endogenous OT stores, mediated partially through glutamatergic mechanisms (Jourdain et al., 1998; Israel et al., 2003), could certainly contribute to the sustained peripheral blood levels seen in IN OT studies. After IN delivery, peripheral OT levels are elevated starting between 10 (Andari et al., 2010) and 30 (Gossen et al., 2012) min and stay elevated for between 150 min (Gossen et al., 2012) to several hours (Burri et al., 2008; van Ijzendoorn et al., 2012), in spite of OTs short plasma half-life (Mens et al., 1983). Vitally, it is currently impossible to distinguish between transnasally absorbed exogenous OT and endogenously secreted OT, so the relative contribution of these two sources to subsequently measured OT levels is unknown. Moreover, though animal studies have demonstrated some concordance between intraneuronal levels of OT and peripheral OT levels (Wotjak et al., 1998; Cushing and Carter, 2000; Wigger and Neumann, 2002), and though several human studies show concordance between peripheral OT levels and naturalistic, centrally mediated behaviors (i.e., parenting, breastfeeding) (Feldman, 2012; Weisman et al., 2012b; for review), the concordance between OT measured in different body spaces (saliva, plasma, CSF) and central effects is still a matter of active debate (Carter et al., 2007; Neumann, 2007).

A variety of important controversies and questions surround the measurement of OT levels. Though space does not permit a full elaboration on the topic, its relevance to many of the questions in this review warrants a discussion. First, we note that there have been controversies regarding the validity and reliability of measurement of OT levels in urine (Anderson, 2006; Young and Anderson, 2010), saliva (Horvat-Gordon et al., 2005), and plasma (Szeto et al., 2011) [and see references in Carter et al. (2007); Szeto et al. (2011); Weisman et al. (2012c); Zhong et al. (2012)]. One aspect of this controversy regards the measurement of OT via immunoassay: the most cost-effective measurement technique for large samples. Notably, a recent report questioned both (1) the accuracy of both radioimmunoassays (RIA) and enzyme immunoassays (EIAs) and (2) the necessity of the technical step of sample extraction which can changes by up to 100-fold the measured levels of the peptide (Szeto et al., 2011). Most recent studies that measure either plasma or saliva OT levels use a commercial OT-Elisa kit (Assay-Design, MI, USA), which has been validated for linearity, cross reactivity, matrix effects, accuracy, precision, and recovery (Carter et al., 2007) though the use of extraction techniques in different studies is variable. In support of the validity of this assay, across a broad range of different studies—including several very large samples (Weisman et al., 2012c; Zhong et al., 2012)—these techniques have produced congruent results, with most of them finding reasonable correlations between saliva and plasma OT levels (Grewen et al., 2010; Feldman et al., 2011; Hoffman et al., 2012), and between OT levels and a wide range of OT-dependent biological processes (White-Traut et al., 2009; Grewen et al., 2010; Feldman, 2012).

A second, related issue surrounding the measurement and meaning of peripheral OT levels is that of endogenous fluctuations in OT levels. Though OT has a diurnal rhythm of daytime rise and night-time decline in mice (Zhang and Cai, 2011) and primates (Amico et al., 1990), the bulk of data does not support significant diurnal variations in plasma OT in humans (Amico et al., 1983; Kuwabara et al., 1987; Challinor et al., 1994; Kostoglou-Athanassiou et al., 1998; Turner et al., 2002; Graugaard-Jensen et al., 2008), [but see Forsling et al. (1998), Landgraf et al. (1982) for evidence of a nocturnal nadir]. CSF levels may differ, as there is some evidence for a diurnal variation in this body space (Amico et al., 1983; Kuboyama et al., 1988). These data on circadian fluctuations stand apart from studies of dynamic fluctuations in OT levels in states like pregnancy (Kuwabara et al., 1987; Fuchs et al., 1992; Lindow et al., 1996), breastfeeding (Jonas et al., 2009; Grewen et al., 2010), orgasm (Carmichael et al., 1987), parenting (Feldman, 2012), and certain stressors (Nussey et al., 1988; Sanders et al., 1990). Also related are documented increases in peripheral OT levels due to both natural variations in estrogen levels (Mitchell et al., 1981; Shukovski et al., 1989) and the ingestion of exogenous estrogen which is known to increase the magnocellular release of OT (Wang et al., 1995) and plasma OT levels (Amico et al., 1981; Silber et al., 1987; Uvnas-Moberg et al., 1989; Michopoulos et al., 2011). Adding complexity is that data on fluctuations in plasma OT levels across the menstrual cycle are mixed, with studies in different healthy and clinical populations showing both variation (Shukovski et al., 1989; Salonia et al., 2005; Liedman et al., 2008) and lack of variation (Stock et al., 1991; Kostoglou-Athanassiou et al., 1998; Light et al., 2005) in normally cycling women, with both estrogen and progesterone levels playing a role.

A third, yoked pair of topics related to OT levels are (1) the correlations between peripheral and central OT levels (see discussion above) and (2) the correlation of OT levels and different disease states. Regarding the latter, investigators have studied OT levels and their relationship to aspects of autism (Modahl et al., 1998; Al-Ayadhi, 2005), eating disorders (Hoffman et al., 2012; Lawson et al., 2012), post-traumatic stress disorder (Seng et al., 2013), schizophrenia (Goldman et al., 2008; Keri et al., 2009; Rubin et al., 2011), social anxiety disorder (Hoge et al., 2008, 2012) and depression (Scantamburlo et al., 2007; Parker et al., 2010). An important but uninvestigated clinical question is whether a transient or chronic increase of peripheral OT levels via treatment with IN OT (Andari et al., 2010; Gossen et al., 2012) correlates with OT-responsive clinical symptoms or treatment-related symptomatic improvement (e.g., whether OT levels may function as a biomarker).

Returning, then, to the clinical issue of OT dose and frequency: though the abovementioned studies of OT levels,—including post-dose OT levels—are somewhat informative regarding the task of determining an optimal OT dose and frequency, to date, there have been no published studies examining the time course of the brain-mediated effects of IN OT, nor of their correlation with peripheral OT levels. Such studies would greatly enhance our ability to optimize OT dose frequency for therapeutic ends. In fact, most studies of IN OT in humans examine its effects at a single time point, typically 30–60 min after administration. For these reasons, in addition to studies examining a range of doses of IN OT, studies examining OT's brain effects over a range of time points are needed to inform optimal OT treatment design.

### Can native oxytocin be improved upon?

Though the native nonapeptide OT has significant advantages in terms of therapeutic modulation of the central OT system, there are problems with peptides. Specifically, neuropeptides like OT lack many “drug-like” properties, especially as regards CNS indications. Though they have certain advantages over other chemical medicinal classes (i.e., evolved specificity for unique functions and receptors, limited drug–drug interactions, little accumulation in tissues, few side-effects), neuropeptides also carry unique liabilities as medications related to their molecular nature (Manning et al., 2012; McGonigle, 2012). These shortcomings include a brief plasma half-life and poor oral bioavailability due to their degradation by plasma and gastric proteases, as well as limited penetrance of the blood-brain barrier due to their large size and hydrophilic nature (McGonigle, 2012).

Technological advances in medicinal chemistry, however, are providing specific solutions to these challenges. In a few cases, medicinal chemists have managed to design small non-peptidergic molecules that bind a specific peptide receptor and are either inactive at that receptor (antagonist) or mimic the actions of the endogenous peptide (agonist). In general this strategy has been much more successful in producing antagonists than agonists (Manning et al., 2012). However, the corpus of preclinical and translation research with OT suggests that it is OT agonists, not antagonists, that have promise as treatments for several psychiatric disorders. With regards to OT, several low-molecular weight non-peptidergic OT agonists have been developed that penetrate the brain after peripheral administration (Pitt et al., 2004; Ring et al., 2010). The only non-peptide OT agonist with experimental evidence for an OT-like behavioral profile is WAY-277464, which had 87% of the binding affinity of OT and significant greater selectivity for the OTR (Ring et al., 2010). Though it exhibited an OT-like anxiolytic behavioral and physiological profile in several animal tests (four-plate test, elevated zero maze, stress-induced hyperthermia), and also an OT-like preclinical antipsychotic profile [reversing amphetamine- and MK-801-induced disruption of prepulse inhibition (PPI)], it did not have an OT-like antidepressant profile [no reduced immobility in the tail suspension test (TST)] (Ring et al., 2010). An interesting corollary finding in this study—one that speaks to the mechanism of action of OT's antidepressant-like effects in the TST—was that a selective OTR antagonist failed to block these antidepressant effects, indicating WAY-27744's effect may be mediated through a different receptor system (i.e., AVPR: also infra vida section “Is Intranasal Delivery of Oxytocin the Optimal Route?”) (Ring et al., 2010) and raising similar questions for OT. In any case, as a result of both pharmacological and market factors, development of WAY-277464 was not pursued by Wyeth (Manning et al., 2012).

Because of the difficulty of developing a non-peptide OT agonist, medicinal chemists have utilized another approach to the problem of stimulating the OT system: chemical modification of the native peptide or an active fragment to increase its resistance to enzymatic degradation and increase metabolic stability. This process has produced carbetocin: an uterotonic OT analog with a peripheral half-life of 85–100 min, significantly longer than OT's (Hunter et al., 1992). Carbetocin—produced by Ferring Pharmaceuticals—is approved in 23 countries outside the United States for post-partum hemorrhage, but there are no published studies investigating its CNS effects in humans. Though a potent uterotonic agent, carbetocin has about 10-fold lower affinity for the OTR than OT (Engstrom et al., 1998; Gimpl et al., 2005), and has been shown to lack anxiolytic efficacy (elevated plus maze) when delivered peripherally, vs. OT, which has anxiolytic efficacy when delivered peripherally (McCarthy et al., 1996; Ring et al., 2006). In another experiment, peripheral carbetocin failed to produce antipsychotic-like effects on PPI (Feifel et al., 2012b). Interestingly, carbetocin did demonstrate an antidepressant-like profile in the forced swim test when administered peripherally and centrally (Chaviaras et al., 2010), and does have short-term anxiolytic effects when delivered centrally (Mak et al., 2012). It would be very instructive to determine carbetocin's effects on centrally mediated processes, especially on clinical symptoms of pyschiatric disorders in humans.

Besides biochemical modifications of the molecules themselves, alternative drug delivery systems (i.e., patches, microspheres, liposomes) can also improve the pharmacokinetic profile of peptides and represent another approach to addressing the challenges of therapeutic modulation of endogenous peptide systems (Patil and Sawant, 2008; Manning et al., 2012; McGonigle, 2012). As an example, a mucoadhesive buccal OT patch has been tested in animals and was able to deliver OT continuously over 3 h (Li et al., 1997). Notably, the efficacy of this or other alternative OT delivery systems on centrally mediated processes has not yet been tested in human or animal studies.

Aside from the delivery of OT or non-peptide OT analogs to the CNS, there are several other ways to impact the central OT system [reviewed in Modi and Young (2012)]. These include inhibition of the non-specific enzyme that degrades OT in the CSF [aminopeptidase placental-leucineaminopeptidase (P-LAP) (Chai et al., 2008; Albiston et al., 2011)] and the use of drugs that may stimulate OT release from endogenous stores via the serotonergic (Jorgensen et al., 2003a) and melanocortin receptors (Sabatier, 2006) found on OT neurons. Drugs like the serotonin 1a agonist buspirone (Bagdy and Kalogeras, 1993; Jorgensen et al., 2003b), 3,4 methlyenedioxymethamphetamine (MDMA) or ecstasy (Thompson et al., 2007; Dumont et al., 2009; Broadbear et al., 2011), and the uniquely effective antipsychotic clozaril (MacDonald and Feifel, 2012a), have been proposed to exert some of their pharmacological activity via stimulation of the central OT system.

### Is intranasal delivery of oxytocin the optimal route?

As mentioned above, a prominent pharmacokinetic issue with synthetic OT involves getting this relatively large, hydrophilic molecule into the brain, given its poor penetration of the blood-brain barrier (McEwen, 2004). IN application of peptidergic drugs to the CNS has been proven since 1989 (Frey, 1991), and is a delivery system increasingly utilized for a variety of drugs for a range of putative central indications, including memory (Benedict et al., 2007) and multiple sclerosis (Ross et al., 2004). Delivering a peptide IN capitalizes first on the heavily vascularized nasal mucosa, which drains through both fenestrated epithelium and via several facial veins (facial and sphenopalatine), into the peripheral circulation, circumventing first pass metabolism (Zhu et al., 2012). In this way, IN delivery simulates IV delivery: drugs delivered IN may reach the brain via active transport or diffusion from the blood compartment across the blood-CSF or blood-brain barrier (Thorne and Frey, 2001; Morimoto et al., 2009). Beyond this, a direct-to-the-brain path of entry after IN delivery of peptides and other drugs has been proposed via two possible mechanisms (Figure 1): (1) intraneuronal active uptake along the olfactory or trigeminal nerve into the brain; and (2) extraneuronal passive diffusion into the CSF through perineural clefts in the nasal epithelium which provide a gap in BBB (Illum, 2004; Thorne et al., 2004; Renner et al., 2012a,b) and (Dhuria et al., 2010; Chapman et al., 2012; Zhu et al., 2012 for references and details).

In point of fact, direct-to-the-brain delivery of IN OT was extrapolated from studies with OT's sister nanopeptide vasopressin, which differs from OT by the substitution of two amino acids (Riekkinen et al., 1987; Born et al., 2002). These studies found that IN delivery increased both CSF levels and plasma levels of AVP (Riekkinen et al., 1987; Born et al., 2002). Indirect support for this hypothesis comes from findings that both vasopressin and OT administered IN have central effects [Fehm et al. (2000) and references therein]. Though contemporary critiques have raised important questions about the details of whether nasal OT gets directly into the brain, and if so, how (Churchland and Winkielman, 2012), support for the direct-to-brain notion comes from recent studies in primates that found that IN OT doubles CSF OT levels within 35 min (Chang et al., 2012), strong evidence of central penetration, given that extant evidence supports that endogenous OT in the CSF derives from central not peripheral sources (McEwen, 2004). Further support comes from recent, unpublished rodent data indicating that IN OT elevates OT levels in the extracellular fluid in the hippocampus and amygdala (Rainer Landgraf, pers. communication). Studies with other IN-delivered peptides showing perineuronal transport are also of interest in this regard (Chen et al., 1998; Renner et al., 2012b; Zhu et al., 2012). On the other hand, increased central levels of OT after IN administration can occur indirectly via elevated levels of OT in the peripheral circulation, thus the evidence described above does not represent definitive evidence of a direct nose-to-brain mechanism, nor whether IN-administered OT has advantages over peripheral or even orally administered OT in terms of brain penetration or reduced peripheral side effects. Indeed, at least one study examining this issue concluded that Devunetide (an 8-amino acid peptide) administered IN to rats entered the brain via the peripheral blood system (Morimoto et al., 2009). As such, given potential disadvantages of IN delivery (i.e., reliance on patients for consistent dose delivery), the issue of whether the IN route or another route is optimal for OT-targeted therapeutics is still an open question.

### What is the role of vasopressin receptors in oxytocin's effects?

Due to their close evolutionary relationship, the pharmacological story of the OT system is interleaved with that of its “sister” hormone AVP. Pivotally, though they have evolved to serve very different functions, these two neuropeptides differ by only 2 amino acids (Gimpl and Fahrenholz, 2001). In terms of receptors, 4 G-protein-coupled receptors have been identified that bind these peptides in both humans and rodents: AVPR1a, AVPR1b, AVPR2, and OTR (Gimpl and Fahrenholz, 2001; Grimmelikhuijzen and Hauser, 2012). Of these receptors, AVPR1a is the most abundantly expressed in the brain, whereas AVPR1b has more limited brain expression and AVPR2 exists almost exclusively in the periphery (Stoop, 2012). Pertaining to OT-directed therapeutics, it is important to note that OT is relatively selective, binding to AVP receptors with ~1% the affinity it binds to OTRs, whereas AVP is non-selective, binding with similar affinity to both OTR and AVPRs (Lowbridge et al., 1977; Mouillac et al., 1995; Manning et al., 2012). Also important is that population-level genetic studies of OT and AVPR1a receptors in humans indicate that both systems appear to be responsible for important behavioral phenotypes in humans (Prichard et al., 2007; Walum et al., 2008, 2012; Levin et al., 2009; Meyer-Lindenberg et al., 2009; Kumsta and Heinrichs, 2013). In terms of conceptualizing their role in mammalian behavior, it appears that differences in receptor co-expression and region-specific density—parameters which vary meaningfully in rodents (see Veinante and Freund-Mercier, 1995; Huber et al., 2005; Young et al., 2006; Raggenbass, 2008), and humans (Loup et al., 1991)—influence the different roles of these two nonapeptides in the CNS, and many models suggest these related peptides have somewhat opposing roles in terms of anxiety and behavioral measures of coping (Legros, 2001; Neumann and Landgraf, 2012). Notwithstanding this larger framework, evidence also exists suggesting some of OTs activities, including both its putative central therapeutic effects (Schorscher-Petcu et al., 2010; Sala et al., 2011), as well as some of its potential side effects [i.e., hyponatremia (Seifer et al., 1985; Stratton et al., 1995)] may be mediated by binding to AVP receptors (Liggins, 1963; Li et al., 2008).

A number of specific agonists and antagonists for each of the four different nonapeptide receptors have been developed (Manning et al., 2012), and have allowed more precise delineation of the role of the different receptors in the activities of each of these nonapeptides. Specifically, whereas OT (but not AVP) normalizes deficits in OT and CD38 knockout mice (Ferguson et al., 2000; Jin et al., 2007), both OT and AVP normalize defects in OT-receptor knockout mice, which demonstrate an autism-like profile (deficits in social behavior, seizures) (Sala et al., 2013). This latter experiment indicated that ICV delivery of both OT and AVP reduced some of these autism-like difficulties via the AVPV1a receptor (Sala et al., 2013). A second, related experiment found that some of OTs analgesic activity in mice is related to AVPV1a, as demonstrated by OT's lack of analgesic activity in AVPR1a knockout mice and the ability of an AVPR1a receptor antagonist to block the effect (Schorscher-Petcu et al., 2010).

With regard to any putative therapeutic effect of OT, OT-mediated activation of AVP receptors may have four potential consequences on OT's behavioral effects: potentiation, mediation, attenuation, or no impact. The same holds true for any possible side effects of OT. Elucidating the role AVP receptor activation plays in each of OT's putative therapeutic effects is therefore important in order to inform development of drugs to optimally target central OT/AVP systems. Knowledge gained from this effort would determine whether energy should be directed toward developing OT agonists with greater selectivity for OTR than OT itself, or toward compounds with more balanced affinity for OTR and one or more AVP receptor types. Highlighting this issue, a recent animal study revealed that AVP1a activation (via desmopressin, an AVP receptor agonist) and blockade (via atosiban, an OT/AVP1a receptor antagonist) produced anxiogenic and anxiolytic effect, respectively (Mak et al., 2012). Based on this, an OTR agonist with no cross-affinity for AVPR1a would be expected to have superior anxiolytic efficacy and a compound that acted as a dual OTR agonist/AVPR1a antagonist might have even greater efficacy. Also worth mention here is a very recent human trial of the vasopressin V1b receptor antagonist SSR149415, which showed negligible anxiolytic effects, and antidepressant effects that warrant further study (Griebel et al., 2012).

### Monotherapy vs. augmentation: can oxytocin treat on its own or is it better suited to augment other established treatments?

The discovery of the molecular mechanisms wherein experience becomes written in the nervous system (Kandel and Squire, 1999), and the growing understanding that OT's effects vary significantly based on context (Bartz et al., 2011b), opens the possibility for pharmacological augmentation of learning-based treatments, including computer-based cognitive training programs (i.e., Vinogradov et al., 2012) and many forms of psychotherapy (see Choi et al., 2010). Several medications have already been examined in this capacity, including the NMDA partial agonist d-cycloserine (DCS) (Otto et al., 2010), the NMDA receptor antagonist ketamine, (Krupitsky et al., 2002), the beta-blocker propranolol (Kindt et al., 2009), and the serotonergic-enhancer MDMA (“ecstasy”), an amphetamine-related CNS stimulant which may exert some of its effects via the OT system (Parrott, 2007; Dumont et al., 2009). Evidence that OT enhances neurogenesis (Leuner et al., 2012), the beneficial effects of social support (Heinrichs et al., 2003), social salience and social memory (Hurlemann et al., 2010; Guastella and MacLeod, 2012) also suggest that OT is a good candidate for such “augmentation” trials. In the case of schizophrenia, for example, it may be valuable to examine OTs effects when given in conjunction with cognitive enhancement therapies already demonstrated to have benefit (Chou et al., 2012; Twamley et al., 2012). Aside from OT's ability to augment learning-based treatments, we note that OT's benefits in schizophrenia have all been when it is given in conjunction with established antipsychotics (i.e., a pharmacological “augmentation” strategy) (Feifel et al., 2010, 2012a; Pedersen et al., 2011; Modabbernia et al., 2013) and that the role of OT as a primary antipsychotic needs investigation.

More speculative, but related, is “OT therapy by proxy” wherein OT given to an adult in a social context (i.e., parent and child) may cause OT-driven changes in the child without direct drug administration to this sensitive population (Naber et al., 2010; Weisman et al., 2012b). Current studies of OT's “augmentation” effect in patients have been limited to one single-dose study and have demonstrated limited success on primary clinical endpoints (Guastella et al., 2009).

### Are there identifiable biomarkers for oxytocin's therapeutic effects?

Biomarkers are objectively measured characteristics that relate to the cause, clinical course, and treatment of illness (Frank and Hargreaves, 2003). In drug development, biomarkers optimize the efficiency of clinical drug studies by facilitating the detection of early signals of drug response (Wiedemann, 2011). Properties of an optimal pharmacological biomarker include: high sensitivity and specificity for clinical outcomes; relatively inexpensive, and low-risk; interpretable by clinicians in many different practice locations; a dose-response relationship; and a plausible link with pharmacology and pathogenesis (Dumont et al., 2005; Wiedemann, 2011; Baskaran et al., 2012; Leuchter et al., 2012). For a variety of reasons—including a chasm between our understanding of the short-term neurobiological effects of drugs and later clinical improvements—these characteristics are of special import in neuropsychiatry research (Wiedemann, 2011). In this field, specifically, a wide variety of biomarkers relevant to OT are in different stages of utilization and development, including laboratory markers (serum markers, genetic tests), electrophysiological markers (EEG, MEG, facial EMG, GSR), brain imaging techniques (fMRI, PET), and behavioral measures (challenge tests, cue exposure tasks, PPI, fear-potentiated startle) (Wiedemann, 2011). Most of these putative biomarkers have been utilized in conjunction with OT, and this is one arena where single-dose OT studies have been invaluable in terms of OT's development as a pharmaceutical. Though there has been relatively little clinically oriented biomarker research with OT (i.e., correlation of a biomarker with meaningful clinical syndromes or outcomes), the extant OT literature contains several promising candidates: heart-rate variability (HRV) (Kemp et al., 2012), skin conductance (GSR) (de Oliveira et al., 2012b), stressed cortisol responses (Ditzen et al., 2009; Quirin et al., 2011; Simeon et al., 2011; Cardoso et al., 2012; Linnen et al., 2012), facial affect recognition (Fu et al., 2007, 2008; Harmer et al., 2009a,b), pupil responses (Leknes et al., 2012), EEG measures (Perry et al., 2010), MEG (Hirosawa et al., 2012), and a variety of functional imaging (fMRI) parameters, including alteration of default-mode network (Sripada et al., 2013), responses to naturalistic social stimuli (Riem et al., 2011, 2012), and stress-induced amygdala responsivity and connectivity patterns (Labuschagne et al., 2010, 2011; Zink and Meyer-Lindenberg, 2012). Regarding functional imaging biomarkers, these techniques would be greatly aided by technical advances, especially a radionucleotide for OTRs. The development of such a tracer—invaluable in the study of clinically relevant aspects of central dopaminergic (Seeman and Tallerico, 1998; Volkow et al., 2001) and opiatergic systems (Greenwald et al., 2003; Mitchell et al., 2012)—would aid in characterizing the relationship between central OTR density, clinical phenotypes, and treatment with OT. *In vivo* visualization of the human OTR would be particularly fascinating given that (1) in animal species, OTR distribution is a significant determinant of behavior (Hammock and Young, 2006; Ross et al., 2009; Ophir et al., 2012); and (2) OTR density appears to vary dynamically during phases of life (Bale et al., 2001; Meddle et al., 2007). As well, functional imaging studies demonstrating the cortical effects of IN OT (Figure 1) and (Bethlehem et al., 2012) are vital additions to translational OT research, given the significant variability of cortical organization among different species, including those most frequently used in OT research (Preuss, 2000). Though a few studies have examined post-mortem OTR binding in the human CNS (using the same radiolabeled peptide as in rodents) (Loup et al., 1989, 1991), synthesis of small-molecule radioligands for the OTR (Smith et al., 2012), would greatly aid our understanding of the functional role of the OT system in human brain disorders and treatment.

To advance the therapeutic potential of OT, the abovementioned biomarkers need to be refined and applied to clinically ill patients. These studies would clarify several basic pharmacodynamics and pharmacokinetic questions surrounding OT (infra supra), and—most importantly—could be used to predict therapeutic response. Vitally, biomarker-guided clinical trials may optimize the efficiency of future clinical trials, facilitating the optimal use of a shrinking pool of funding for OT research (driven in part by OT's lack of patent exclusivity).

## From dearth to birth, and preclinical to clinical research—helping oxytocin deliver

We believe the above review supports two broad conclusions about OT as potential therapeutic agent for CNS disease. First, the last decade of translational and clinical research has provided a great deal of reason to be cautiously optimistic that OT-based treatments may be developed to help ease the dearth in novel treatments for psychiatric illness. Secondly, and somewhat in contrast, the translation of OT's therapeutic promise has been remarkably slow, considering clinical studies with OT are not hindered by the typical limitations imposed by non-approved investigational drugs (i.e., costly animal and human safety and toxicity testing before testing in proof-of-concept human trials). As discussed above, single-dose studies in normal subjects—and a much-smaller set of single-dose studies in clinical populations (Table 1)—has left the field pregnant with anticipation about OT's potential therapeutic utility. In our opinion, however, direct tests of this utility are now past due. We need to help OT deliver.

The fact that there are only a few published small, multi-week clinical trials of OT is problematic. More single-dose studies—overwhelmingly in normal subjects—continue to be generated. Some of these add to the body of support for therapeutic effects, while others do the opposite, revealing a more complex role for OT in human behavior, emotion, and cognition (De Dreu et al., 2010, 2011). These complexity-revealing findings in particular have spurred some investigators to suggest that it is premature to speculate about OT's therapeutic potential for neuropsychiatric disorders and opine that before we do clinical trials, the field needs more translational studies to elucidate OT's complex role (e.g., Grillon et al., 2012; Miller, 2013).

As active clinicians and translational researchers, we recognize the value of translational research. Faced daily with individuals and families who have profound and often urgent need for better treatments, however, we also recognize its limitations. While we agree that additional preclinical OT research in animals and humans is vital, we do not believe these trials should be done at the expense of randomized controlled trials in clinical populations. Instead, a stepwise, tandem progression is optimal. Translational research works best in a bi-directional mode, with preclinical studies informing clinical trials and the results of clinical trials—in turn—helping identify which preclinical paradigms have the best predictive validity for a specific disorder and drug class. In this way, translational paradigms can be further leveraged to conduct impactful preclinical research. Animal studies have the ability to efficiently deliver clinically relevant information without the expense, time, and risk-considerations inherent in human trials. Similarly, preclinical human studies in which acute effects of drugs, like OT, are examined on symptom proxies such as functional imaging changes in clinically relevant circuits or laboratory analogs of pathological conditions (e.g., CCK-induced panic) are much easier, less expensive, and less risky than classic randomized clinical trials. However, at present, the predictive validity of both these forms of preclinical drug research with regard to psychiatric disorders is far from perfect. Examples abound in which efficacy or deleterious effects noted in clinical trials were not manifested in preclinical studies and vice versa. Though clearly we support translational research and the ongoing search for reliable biomarkers of clinical response in psychiatric illness, the simple facts are: rodents are not humans, and changes in functional imaging or laboratory tasks are not the same as changes in clinical symptoms.

As one can see in the abovementioned review, there is currently reasonably good evidence from animal and human preclinical trials that OT may have therapeutic benefit in at least three brain-based conditions: schizophrenia, anxiety, and autism. For the myriad reasons discussed above, we believe that additional preclinical studies will not—by themselves—answer the critical therapeutic questions that face clinicians in the field. Therefore, proof-of-concept clinical trials are warranted. As a point of comparison, phase II studies—the kind that are now needed to directly test the various hypotheses regarding OT's therapeutic utility—have been carried out by industry on investigational agents with far less data supporting their efficacy and safety. These facts, together with (1) the profound impairments imposed by brain-based disease; (2) the often-inadequate efficacy of extant treatment; and (3) a disheartening lack of promising, novel treatments in the pipeline, we believe, justifies cautious execution of clinical trials with OT in the abovementioned conditions. In this regard it is noteworthy that—after a long period of gestation—the water seems to have broken on research directly testing OT's clinical promise: several potentially seminal clinical studies of OT appear be underway in several top “target” disorders (www.clinicaltrials.gov).

However, OT will not be developed into a drug for any psychiatric indication with one, two, or even three investigator-initiated clinical trials. These initial trials often produce negative or weak therapeutic effects. Thus, concurrent preclinical studies—both animal and human—are needed to advance the therapeutic development of OT. These additional preclinical studies are needed to inform the design of the inevitable second wave of clinical trials in the current “big three” indications, as well as initial proof-of-concept trials in other emerging candidate OT-responsive disorders. For example, preclinical studies demonstrating dichotomous dose-dependent effects on clinically relevant measures may prompt research emphasis on wider dose ranging in phase-II studies of OT. Likewise, animal studies showing tolerance of clinically relevant preclinical effects after a certain duration of treatment may prompt longer-duration trials to evaluate this effect in humans. Similarly, evidence of adverse effect in animals emerging at certain doses or durations may prompt incorporation of specific safety monitoring features into OT clinical trials. This latter point is particularly important given that randomized controlled trials are costly and labor-intensive, and that negative results due to type-II errors (i.e., missing a significant therapeutic effect) for example, by infelicitous selection of dose(s) or dosing frequency can be devastating to future studies.

To this end, there is a definitely a need for more translational research using animal models with validity—particularly predictive validity—for the specific conditions for which OT is a candidate treatment (e.g., autism, schizophrenia, anxiety, etc.). Such studies will help address the vital questions we have delineated in this paper. These animal studies should be complemented by translational human studies using single doses and non-symptom outcomes. Knowledge derived from both of these approaches will increase the likelihood of success of critical clinical trials. As mentioned above, the third element in the bench-to-beside arc are clinical trials using OT, which can reciprocally provide useful information to evaluate the predictive validity of various animal models and proxy-symptom human paradigms.

In light of these facts, and in light of the prodigious amount of animal and human OT research, it is surprising how little effort has been specifically directed to address the translational questions delineated above. For example, despite good evidence from preliminary clinical trials that OT has therapeutic benefit in schizophrenia (MacDonald and Feifel, 2012a), at the time of this writing, only three published studies have explored exogenous OT's effects in animals models with predictive relevance specifically for schizophrenia (Feifel and Reza, 1999; Lee et al., 2005; Feifel et al., 2012b). In order to help OT deliver on its therapeutic promise, there remains much work across the entire translational spectrum.

### Conflict of interest statement

David Feifel is a named inventor on a method of use patent for oxytocin submitted by UCSD. K. MacDonald declare that the research was conducted in the absence of any commercial or financial relationships that could be construed as a potential conflict of interest.
